# Anesthesia for Pars Plana Vitrectomy with Insulin Needle, Is It Possible?

**DOI:** 10.1155/2012/179768

**Published:** 2012-08-08

**Authors:** Waleed Riad, Nauman Ahmed, Emad Abboud, Essam Al-Harthi, Eman Kahtani

**Affiliations:** ^1^Anesthesia Department, Toronto Western Hospital, University Health Network, Toronto, ON, Canada M5T 2S8; ^2^Anesthesia Department, King Khaled Eye Specialist Hospital, Riyadh 11462, Saudi Arabia; ^3^Vitreoretinal Division, King Khaled Eye Specialist Hospital, Riyadh 11462, Saudi Arabia

## Abstract

Peribulbar block is commonly used for ocular posterior segment surgery. This work aimed to compare the efficacy of using 12.5 mm to 25 mm standard needle length in performing single injection peribulbar block for retinal surgery. Peribulbar block was performed in 120 patients using either standard 25 mm or 12.5 mm 30 G needle (insulin needle). While applying digital pressure around the needle hub, 8–10 mL of local anesthetic are injected. Ocular movement was assessed at 5 and 10 min using simple akinesia score (0–8). If after 10 min score was >1, supplementary injection was given. Visual analogue scale (0–10) was used at the end of the procedure to assess surgeons' satisfaction and patients' intraoperative pain. No differences in akinesia score at 5 & 10 min (*P* = 0.34 and 0.36, resp.). Initial volume injected was comparable between groups (*P* = 0.31), however total volume of local anesthesia and supplementary injections were significantly higher in 12.5 mm group (*P* = 0.03 and 0.01, resp.). No difference as regard surgeons' satisfaction and patients' intraoperative pain (*P* = 1.0 and 0.18, resp.). Peribulbar block with 12.5 mm needle together with digital compression is a suitable alternative to the standard block with 25 mm needle length for retinal surgery.

## 1. Introduction

Regional anesthesia is standard technique for most ophthalmic procedures including ocular posterior segment procedures. In many parts of the world, retrobulbar anesthesia has almost been replaced by peribulbar technique because of higher margin of safety and fewer side effects. The advantages of this approach also include less disruption to the patient's physiology, rapid return to normal routines, and better utilization of hospital beds and economy [1]. Peribulbar anesthesia that consists of introducing a needle into the extraconal space should theoretically be safer than retrobulbar anesthesia as it is away from the vessels and optic nerve. It is based on the principle of tissue compartments. When a needle is inserted in one compartment and the local anesthetic injected, it will spread under the effect pressure and volume to other compartments. Therefore, the local anesthetic injected into the extraconal space should spread to the intraconal space to provide anesthesia and akinesia of the globe [2].

From the time when peribulbar block was first described by Davis and Mandel, it has undergone consistent improvements and refining [3]. Historically, Bloomberg described a technique where the anesthetic solution is deposited more superficially outside the muscle cone, approximately 18 mm from the skin surface (anterior peribulbar block). Five mL of anesthetic agent is injected into the superonasal orbit and a further 5 mL inferotemporally [4]. A small volume peribulbar block combined with block of the facial nerve at the lateral orbital rim has been encouraged to minimize chemosis of the surface tissues [5]. Some authors have found a single peribulbar injection either through the lower lid [6] or the upper lid [7] just as effective and safe.

Another important modification to the original technique is change of the needle length used to deliver the block. Many authors believe it is an important consideration for the safe conduct of the technique. Globes longer than 26 mm are particularly at risk [8]. Patients who are presenting for retinal detachment surgery are likely to have long eyes. This is of great importance especially in a situation where B-scan is not done. For intraconal and extraconal injections, shorter 25 mm needles are recommended. Moreover, many researchers have shown excellent results with 5/8-inch needle [9, 10]. Previously, the authors demonstrated efficiency of 12.5 mm, 27 G needle for anterior segment procedures [11]. However, posterior segment surgeries required solid block with adequate akinesia as there is excessive globe manipulation and the surgical procedure required more time compared to anterior segment surgery. To the best of our knowledge, there was no previous studies described the usage of 12.5 mm length needle (insulin needle) for posterior segment surgeries. The aim of this randomized control trial was to compare the efficacy of using 12.5 mm to 25 mm standard needle length in performing single injection peribulbar block through the inferotemporal approach for patients undergoing Pars Plana Vitrectomy. 

## 2. Method

One hundred and twenty patients undergoing Pars Plana Vitrectomy (PPV) under regional anesthesia were enrolled in this prospective randomized double-blinded study, after approval of the hospital Research and Human ethics committees, and informed patient written consent, were obtained. Regional block was performed using a disposable insulin needle of 30 G and 12.5 mm needle length (Becton Dickinson, BD Microlance 3, Benelux, Belgium). Anesthesia was performed by either of the anesthesiologists investigators (WR or NH) involved in the study. Exclusion criteria include patients allergic to local anesthetic solutions; with local sepsis, serious impairment of coagulation, and orbital abnormalities; or who were unable to cooperate in maintaining a relatively motionless supine position; or who refused the anesthetic technique.

On the day of surgery, patients were requested to fast 6 hours and premedicated with hydroxyzine 0.5–1.5 mg kg^−1^, paracetamol with codeine (Revacod) 10–15 mg kg^−1^ one hour before surgery according to the standard practice of the institute. On arrival of the patient in the preoperative holding areas, baseline globe movements in the major directions of gaze (superior, inferior, medial, and lateral) were assessed. Standard monitoring of ECG, pulse oximetry, and noninvasive blood pressure were started and venous access was initiated.

Patients were randomly allocated using a computer generated numbers and sealed envelope to either group. Peribulbar block performed through insertion of the standard 25 gauge, 25 mm needle length (Group 1), or 30 gauge, 12.5 mm needle length (Group 2). The 12.5 mm needle was initially designed for administration of subcutaneous insulin or tuberculin test. In both groups, the needle inserted through the lower eyelid as far lateral as possible in the inferotemporal quadrant, just above the inferior orbital notch. It is inserted perpendicularly to the skin until the hub rested on the inferior orbital bony rim. This typically allows fixation of the needle in position and prevent its migration backward during injection. Digital pressure was applied by the thumb and index fingers around the needle hub during injection. This prevents needle from further displacement and promotes a posterosuperior spread of injectable instead of accumulation into the lower eyelid. After negative aspiration,a volume of 8–10 mL of local anesthetic solution of Bupivacaine 0.5%, Lidocaine 2% in a ratio of 3 : 2 (Astra, Astra Sodertalje, Sweden) with hyaluronidase 5 unit mL^−1^ (CP Pharmaceutical Ltd, Wrexham, United Kingdom) was injected until total drop and fullness of the upper eyelid. No pressure reducing device (Honan's balloon) was used, however intermittent digital message of the eye was applied. Ocular movements were assessed 10 minutes after the block. A simple akinesia scoring system was used [12]. Eyes movement in four directions is elicited superiorly, inferiorly, nasally, and temporally. A normal movement score two, partial movement score one, and flicker or no movement score zero. Merging the four directions of ocular movement, a maximum score of eight and a minimum score of zero could be achieved. Using this scale, an ocular mobility was assessed at five and 10 min after block by an observer who was unaware about the needle used for block. A score of one or zero was accepted to allow the surgeon to operate. If after 10 min, the required degree of block was not achieved, a supplementary anesthesia was provided. This means addition of 3–5 mL anesthetic solution either medially if the movement is medial or inferior or superiorly if the movement is lateral or superior using the same needle used for the primary block. Surgeons who were blind to the technique used were asked to rate their satisfaction with the anesthetic technique using a scale from 0 (total dissatisfaction) to 10 (total satisfaction). Similarly at the same time, Visual analogue scale was used to assess the patients' level of pain (0 = no pain to 10 = worst pain ever). Any complication and additional supplementation of local anesthetics were documented.

### 2.1. Statistical Analysis

The results were analyzed using the Statistical Package for Social Science for Windows version 14 (SPSS Inc., Chicago, IL, USA). Sample size was calculated using N-Query software version 4, it indicated that 60 subjects were required in each arm to detect a difference of 0.5 in the mean of simple akinesia score which is the primary objective when *α* error equals 0.05 and *β* error equals 0.20. Numerical data were analyzed using independent sample, two-tailed *t*-test, and *e* expressed as a mean and standard deviation (SD). On the other hand, categorical data were analyzed by chi-square test and expressed in number and percentages. *P* value of 0.05 was used as the level of significance.

## 3. Results

One hundred and twenty patients were enrolled in this prospective randomized double-blinded study. Demographic and descriptive data are shown in Table 1. No difference between groups observed as regard age, weight, height, sex, ASA classification and duration of surgery.

Clinical data are shown in Table 2. There was no statistical difference between the two group as regard akinesia score at 5 and 10 minutes after block (*P* = 0.35 and 0.36, resp.). There was no significant difference for the initial volume of inferotemporal injection (*P* = 0.31), however total volume of injectable after supplementary injections was significantly higher in 12.5 mm group (*P* = 0.03). Additionally, number of patients required supplementary injections were significantly higher in 12.5 mm group (*P* = 0.01). There was no difference between groups as regards surgeons' satisfaction with the block as well as patient's experience with intraoperative pain (*P* = 0.75 and *P* = 1.00, resp.).

In this study, no major life threatening complications were recorded. There were no cases of retrobulbar hemorrhage, globe perforation, central spread, optic or retinal damage and periocular hematoma.

## 4. Discussion

The current work showed that posterior segment surgery could be performed with insulin needle. The orbit could be divided into three spaces (anterior, mid, and posterior) for better understanding of the relationship of injection site. The anterior orbit ends 2–5 mm anterior to the equator of the globe and is filled primarily with connective tissue [13]. Insertion of longer needles deep into the orbit increases the potential of injury to important structures and limiting the depth of needle insertion may limit needle injury [14]. In an interesting study, Scott and collaborators clearly reported that a 16 mm needle reaches to the junction between anterior and mid orbit and cannot pass beyond [9]. It is logical the 12.5 mm needle length will rest only in the anterior orbit if it was not pushed posteriorly. If globe perforation happened by the short needle. If inadvertent globe perforation and/or penetration occurred by the short needle the consequences should be less severe than longer needle as it does not involve the optic nerve, fovea, and ophthalmic artery located in the posterior orbit.

It had been documented that akinesia is used as a primary measure of block effectiveness [11]. In the current work we tighten our score to one or zero compared to the commonly accepted score of three. This is because of the nature of the surgery that is lengthier and generates more pain compared to anterior segment surgery. Inspite of that, the required score was achieved in all patients. However, 12.5 mm group required more volume and higher number of supplementation. This could be explained by the distance, which the injectable needs to travel till it reaches its target area. In agreement with our data, Ripart and colleagues who demonstrated that local anesthetic injected extraconally has a longer way to spread into the cone to block all nerves responsible of the sensory, motor, and autonomic innervations of the eyeball. Increasing the volume injected will accomplish this need for more local anesthetic spread [2].

Regional block for posterior segment surgery facilitates rapid turnover, as patients usually blocked ahead of time in the holding area. Knight and associates reported in their audit that retinal surgeons were satisfied to operate on patients who had peribulbar block provided that they had good akinesia and adequate analgesia [15]. In the same audit, 96.6% of patients were totally satisfied with the technique. Our work prove that both surgeons and patients were equally satisfied with the standard as well as short needle peribulbar block.

## 5. Conclusion

Using 12.5 mm needle length (insulin needle) for peribulbar blockade showed agreeable results compared to the standard 25 mm needle length. This technique is a suitable effective alternative for posterior segment surgery.

## Figures and Tables

**Table 1:** Demographic and descriptive data.

	Group 1	Group 2	*P* value
	(25 mm)	(12.5 mm)	
	(*N* = 60)	(*N* = 60)	
Age (years)	59.7 (8.5)	62.0 (9.5)	0.13
Weight (kg)	80.6 (23.4)	75.4 (16.8)	0.16
Height (cm)	158.3 (21.3)	159.9 (16.2)	0.31
Sex			
Male	36 (60%)	39 (65%)	0.32
Female	24 (40%)	21 (35%)	
ASA grade			
1	3 (5%)	2 (3.3%)	0.35
2	13 (21.7%)	15 (25%)	
3	44 (73.3%)	43 (71.7%)	
4	0	3 (5%)	
Duration of anesthesia (min)	122.23 (34.4)	119.73 (43.6)	0.70

Data expressed as a mean value and standard deviation or number and percentages.

**Table 2:** Clinical data.

	Group 1	Group 2
	(25 mm)	(12.5 mm)
	(*N* = 60)	(*N* = 60)
Akinesia score		
5 min	0.90 (1.2)	0.67 (1.1)
10 min	1.12 (1.2)	0.85 (1.1)
Initial volume injected (mL)	9.47 (0.9)	9.63 (0.8)
Total volume injected (mL)	11.03 (3.2)	12.52 (4.1)*
Patients required supplementary injections	16 (26.7%)	28 (46.7%)*
Surgeons' satisfaction	9.35 (0.56)	9.39 (0.51)
Intraoperative pain (VAS 0–10)	0.58 (1.3)	0.31 (0.8)

Data expressed as a mean value and standard deviation or number and percentages.

*P value < 0.05.
